# Hydrodynamic characteristics of submerged vegetation flow with non-constant vertical porosity

**DOI:** 10.1371/journal.pone.0176712

**Published:** 2017-04-27

**Authors:** Mingdeng Zhao, Zilong Fan

**Affiliations:** State Key Laboratory of Water Resources and Hydropower Engineering Science, Wuhan University, Wuhan, Hubei, China; Coastal Carolina University, UNITED STATES

## Abstract

In order to investigate the influence of the vertical variation of porosity on open-channel flow with submerged vegetation, vertical non-homogeneous stumps and stems in submerged vegetation flow are simulated with truncated cones in a laboratory flume. First, porosity is defined as a function of water depth. A new governing equation for vegetation flow is established on the basis of the poroelastic media flow theory, and its analytical solution is obtained with the finite analytic method. Then, the fitting expression of permeability is established with experimental data, which shows the variation in permeability with vertical porosity and vegetation density. Finally, the calculated velocity distribution is compared with the measured velocity distribution. The theoretical results are in good agreement with the experimental data, which indicates that the theoretical formula accurately and practically predicts vertical velocity distribution in complex open-channel flow with submerged vegetation.

## Introduction

Vegetation flow generally exists in nature, and plays a significant role in flood control and sediment transport. In addition, Aquatic plants can purify sewage and provide habitat for microorganism and aquatic animals, which is beneficial to the river ecosystem. Therefore, the research of vegetation flow has become a focus in environmental hydraulics.

Previous scholars have made numerous achievements on open-channel flow through rigid vegetation, but they assumed vertical porosity to be constant during calculation and selected rigid cylinder to simulate vegetation in their experiments. For example, Ghisalberti M. and H. M. Nepf [1] applied a one-dimensional numerical model to predict the vertical velocity distribution of submerged vegetation flow by assuming a single mixing length above the vegetation. The model was verified with experimental data. Cui J et al. [2] investigated fully developed turbulent flows with submerged vegetation by using Large Eddy Simulation. In addition, their study analyzed the role of coherent structures on the momentum transfer across the water-plant interface. By placing a typical cylindrical stem in the middle of a vegetation zone, Kothyari et al. [3] used strain gauge to directly measure drag force. To investigate the effects of vegetation on flow structure, Gao. G et al. [4] applied a model with the two-layer mixing length turbulence closer to a physical model channel. Based on the Navier-Stokes-Forchheimer equation, Guo J and Zhang J [5] focused on velocity distributions for laminar and turbulent flow through emergent and submerged vegetation. Their study reported that brief Jacobi elliptical functions can exactly describe laminar flow through both emergent and submerged vegetation, whereas turbulent submerged vegetation flow was approximated by a hyperbolic sine law. Recently, some scholars paid attention to the influence of spatial variation of vegetation on channel flow. For example, Ricardo et al. [6] measured flow in a channel with spatially varying distribution. The variation was achieved by changing longitudinally the stem area number density and stem distribution. But they still did not consider the varying vertical porosity. In nature, practical porosity varies vertically with stem thickness and the leaf density. If a circular cylinder is selected to simulate rigid vegetation in the numerical simulation and experiments, the research results may cause deviation in practical engineering.

Many previous researchers [7–9] modeled vegetation flow in channels as porous media flow because of the similarity. For example, Hsieh et al. [9] investigated the vertical velocity profile of flow passing over a vegetal area by applying Boit’s theory of poroelasticity and discussed five factors’ effect on vegetation flow. In their researches, permeability is the most important factor in affecting flow characteristics. However, there was not an accurate expression of permeability in submerged vegetation flow. Recently, Xu et al. [10] have summarized some modifications of the permeability and presented an analytical expression for the permeability based on the fractal characters of porous media and capillary model. Nevertheless, none of them can be used in submerged vegetation flow directly.

In order to obtain more practical results, a truncated cone is selected to simulate rigid vegetation to explore the influence of the varied vertical porosity on submerged vegetation flow. A three-layer model (upper free water layer, interface layer and vegetation layer) for submerged vegetation flow with variation of vertical porosity is proposed to predict vertical distribution of velocity. The governing equation for velocity in the whole area of submerged vegetation flow is presented by applying the poroelastic media flow theory [11, 12]. The fitting expression of permeability *k* in submerged vegetation flow is also obtained from experimental data. With the finite analytic method, the new finite analytic solution for velocity in the vegetation layer and the interface layer is presented with high accuracy. Furthermore, the calculated velocity distribution agrees with experimental data in a flume experiment. The model and approach presented in this paper can predict velocity distribution of submerged vegetation flow in river ecological restoration. Even if aquatic plants have complex shapes, the velocity distribution can be obtained accurately with the finite analytic method because of considering the influence of varying vertical porosity on vegetation flow. Therefore, this research provides a new vision for researching submerged vegetation flow in a complex environment.

## Variation of vertical porosity

**Fig 1:**
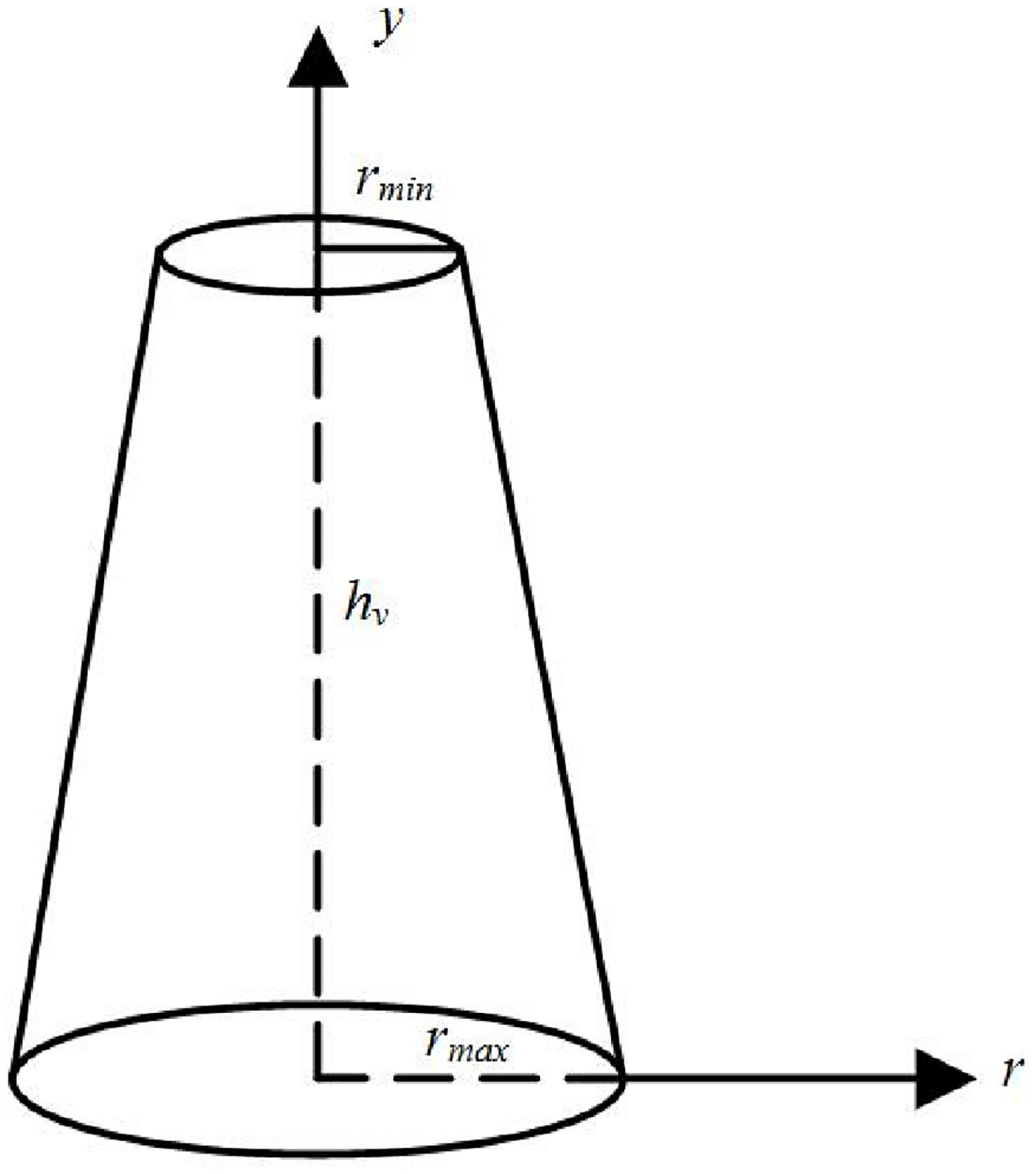
Relationship between vegetation height and sectional radius.

For a truncated cone is selected to simulate rigid vegetation in submerged vegetation flow, sectional radius *r* is a function of vegetation height *y* as shown in Fig 1.

The linear equation is as follows:

r(y)=ay+b (1)

Vegetation porosity is a function of plant shape, number of plants per unit area, and vertical variation of stem thickness. For circular sections, the vegetation porosity *n* is obtained as:

$$n\left(r\right)=1-\alpha \pi r^{2}$$ (2)

where *α* is the number of plants per unit area. Inserting Eq (1) into Eq (2) yields the following expression between vegetation porosity and height:

$$n\left(Y\right)=c_{1}Y^{2}+c_{2}Y+c_{3}$$ (3)

where *h* is the total water depth, *c*_1_ = −*απα*^2^*h*^2^, *c*_2_ = −2*απαbh*, *c*_3_ = 1 − *απb*^2^, and $Y=\frac{y}{h}$. Vegetation is a function of the dimensionless water depth at a given water depth, number of plants per unit area, and stem shape.

## Governing equation and boundary conditions

### Governing equation

Flow is assumed to be uniform and steady, and that vegetation is rigid and will not move and deform. Applying Biot’s poroelastic theory [11, 12] to submerged vegetation flow yields the continuity equation below:

$$\frac{\partial nu_{j}}{\partial x_{j}}=0$$ (4)

The momentum equation is as follows:

$$\rho g_{i}-\frac{1}{n}\frac{\partial np}{\partial x_{i}}+\frac{\mu }{n}\left\{\frac{\partial }{\partial x_{i}}\left[n\left(\frac{\partial u_{j}}{\partial x_{i}}+\frac{\partial u_{i}}{\partial x_{j}}\right)\right]-\frac{n^{2}}{k}u_{i}\right\}=\rho \frac{\partial u_{i}}{\partial t}+\rho u_{j}\frac{\partial u_{i}}{\partial x_{j}}$$ (5)

where *i*, *j* = 1, 2, 3. The variable *u*_*i*_ is the velocity component in the *x*_*i*_ − direction, *g*_*i*_ is the acceleration component of gravity in the *x*_*i*_ − direction, and *u*_*j*_ is the velocity component in the *x*_*j*_ − direction. *μ* is viscosity of fluid, *p* is fluid pressure, *ρ* is fluid density, and *k* is the special permeability of porous media.

For a truncated cone selected to simulate rigid vegetation in submerged vegetation flow, vegetation porosity is not constant but is a parabolic function of dimensionless depth. Therefore, porosity *n* in Eqs (4) and (5) is not negligible. The governing Eq (5) for turbulent flow is simplified to:

$$n\mu \frac{d^{2}u}{dy^{2}}+\mu \frac{\partial n}{\partial y}\frac{\partial u}{\partial y}+\frac{d\left(-n\rho \bar{u^{,}v^{,}}\right)}{dy}-\frac{\mu n^{2}}{k}u+n\rho gs=0$$ (6)

where $-n\rho \bar{u^{,}v^{,}}$ is Reynolds shear stress *τ*_2_. Applying a turbulence model for the relation of Reynolds stresses in turbulence theory yields the parabolic eddy viscosity model expressed as:

$$\tau_{2}=-n\rho \bar{u^{,}v^{,}}=n\beta \rho \sqrt{s}{\left(\frac{y}{h}\right)}^{2}\frac{du}{dy}$$ (7)

where *s* is the channel bed slope, *β* is the coefficient of turbulence, and the unit of *β* is m^2^/s. In submerged vegetation flow, *β*_1_ is the coefficient of turbulence in the vegetation region and *β*_2_ is the coefficient in the water region.

By substituting Eq (7) into Eq (6), the governing equation for turbulent flow can be described as

$$\left[1+\frac{\beta \rho \sqrt{s}}{\mu }{\left(\frac{y}{h}\right)}^{2}\right]\frac{d^{2}u}{dy^{2}}+\left(\frac{1}{n}\left[1+\frac{\beta \rho \sqrt{s}}{\mu }{\left(\frac{y}{h}\right)}^{2}\right]\frac{dn}{dy}+\frac{2\beta \rho \sqrt{s}}{\mu h}\frac{y}{h}\right)\frac{du}{dy}-\frac{n}{k}u+\frac{\rho gs}{\mu }=0$$ (8)

which can be rewritten in the dimensionless form:

$$(1+\zeta Y^{2})\frac{d^{2}U}{dY^{2}}+\left[\frac{1}{n}(1+\zeta Y^{2})\frac{dn}{dY}+2\zeta Y\right]\frac{dU}{dY}-\lambda U+2=0$$ (9)

where $\zeta =\frac{\beta \rho \sqrt{s}}{\mu }$, $U=\frac{2\mu u}{\rho gh^{2}s}$, and $\lambda =\frac{nh^{2}}{k}$.

### Boundary conditions

**Fig 2:**
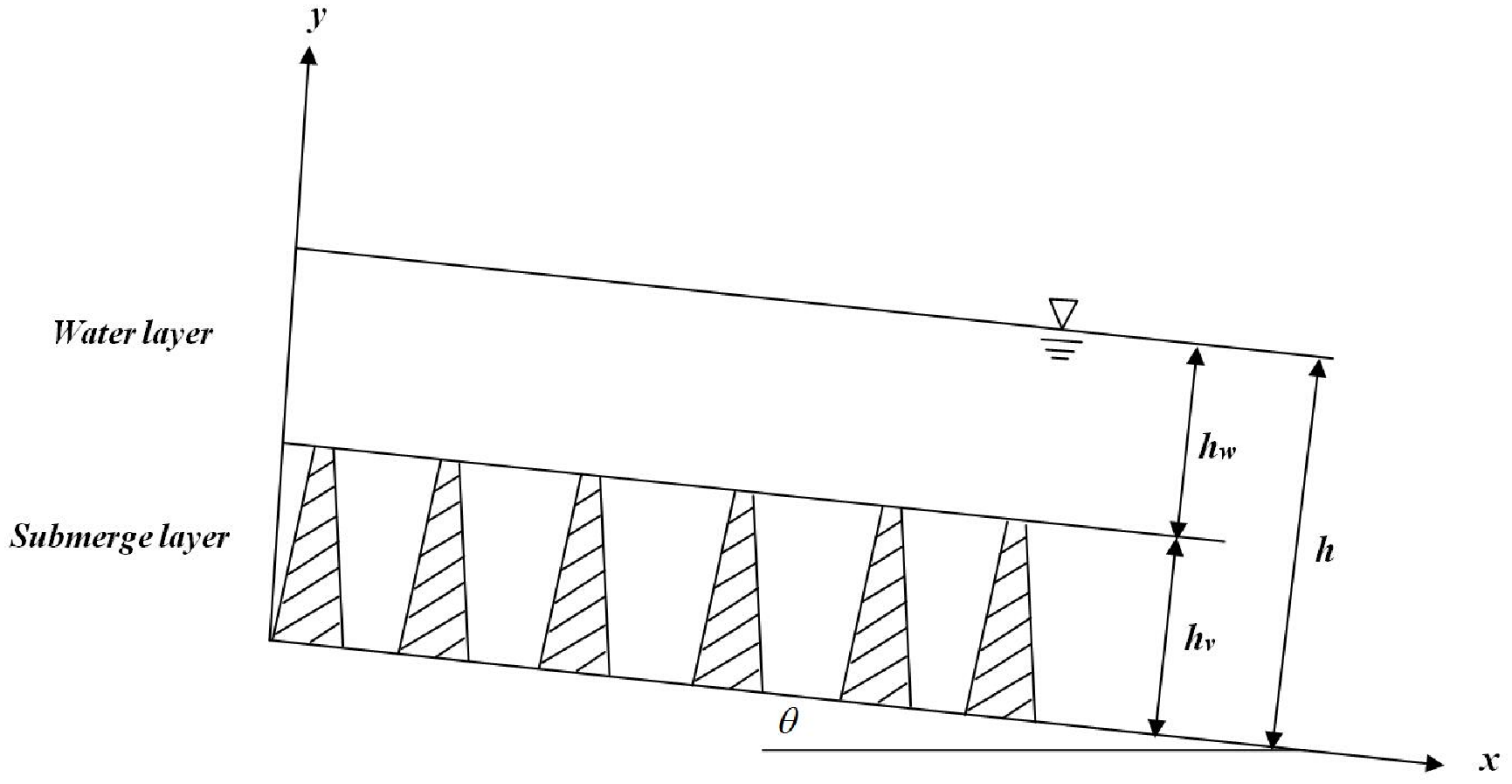
Uniform wetland flows with submerged vegetation.

According to Deresiewicz and Skalak [13], the boundary conditions of submerged vegetation shown in Fig 2 can be obtained as follows:

(1) At the free surface (*y* = *h*, *Y* = 1)

$$\frac{dU}{dY}=0$$ (10)

(2) At the interface between the water layer and the vegetation layer (*y* = *h*_*v*_, *Y* = *H*_*v*_)

$$\frac{dU_{-}}{dY}=\frac{dU_{+}}{dY}$$ (11)

$$nU_{-}=U_{+}$$ (12)

where *U*_+_ is the boundary velocity in the water layer, *U*_−_ is the boundary velocity in the vegetation layer, *h*_*v*_ is the height of vegetation layer, and *H*_*v*_ = *h*_*v*_/*h*.

(3) Assuming an impermeable bed, the non-slip condition gives

U=0 (13)

## Finite analytic solution

(1) In the water layer (*H*_*v*_ ≤ *Y* ≤ 1)

For the water layer (*n* = 1), Eq (9) can be simplified to:

$$(1+\zeta Y^{2})\frac{d^{2}U}{dY^{2}}+2\zeta Y\frac{dU}{dY}+2=0$$ (14)

Thus, the analytical solution of velocity is obtained by solving Eq (14):

$$U(Y)=A-B\frac{\text{arctan}(\sqrt{\zeta }Y)}{\sqrt{\zeta }}-\frac{\text{ln}(\zeta Y^{2}+1)}{\zeta }$$ (15)

where *A* and *B* are the coefficients of the analytical solution. They can be determined by boundary conditions.

(2) In the vegetation layer with non-constant porosity

Chen et al. [14] presented the finite analytic method for solving partial differential equations. The basic idea of the finite analytic method is the incorporation of the local analytic solution in obtaining the numerical solution. The finite analytical method first divides the total region of the problem into small sub-regions in which local analytic solutions are obtained. Then an algebraic equation is derived from the local analytic solution for each sub-region relating an interior nodal value at a point in the sub-region to its neighboring nodal values. The assembly of all the local analytic solutions thus provides the finite-analytic numerical solution of the problem.

**Fig 3:**
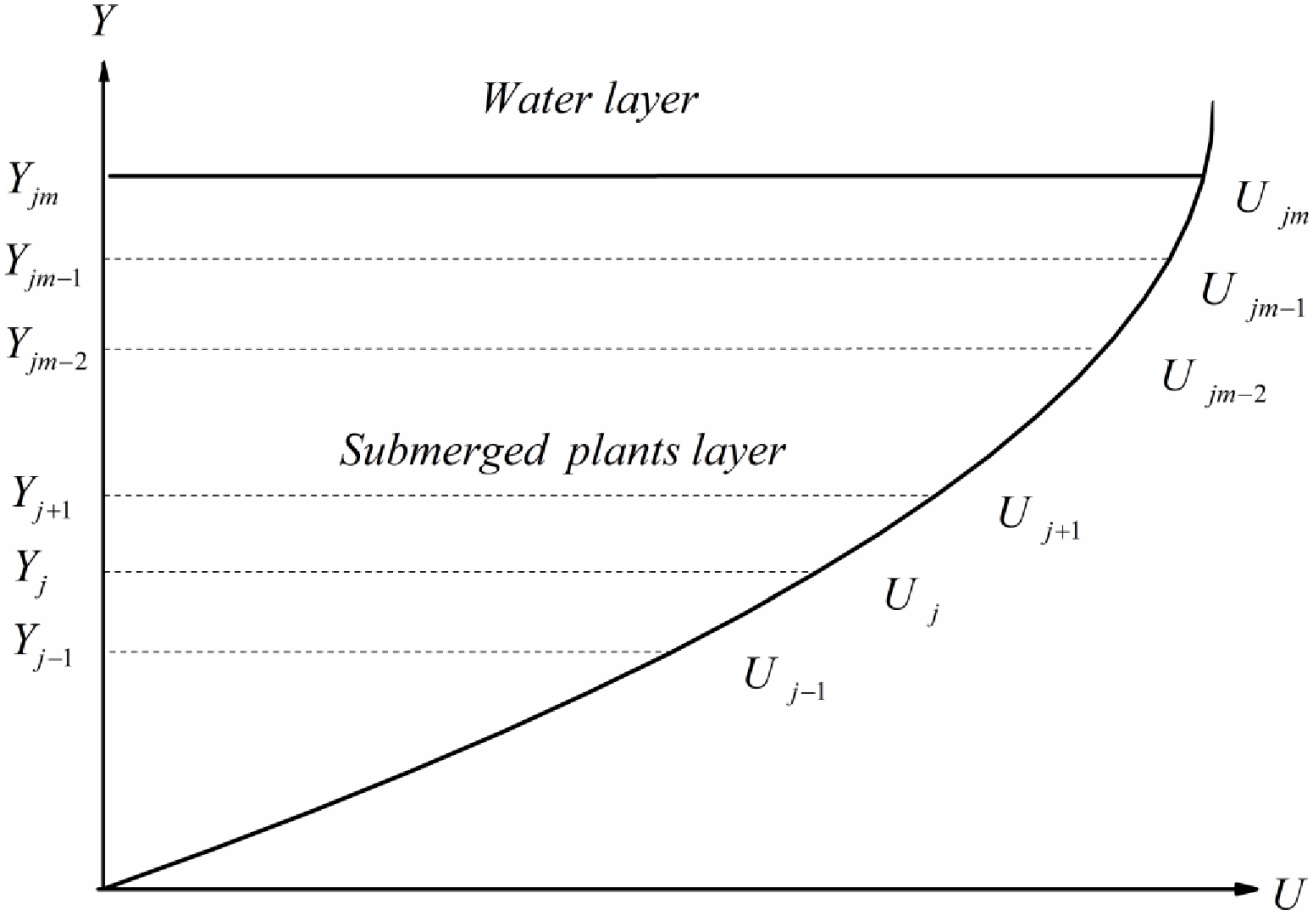
Sketch of the finite analytic method.

When vegetation porosity varies vertically with water depth, Eq (9) cannot be solved directly with the modified Bessel function, because it is the second order nonlinear differential equation. In order to analytically obtain the solution, the vegetation layer is divided into several small regions, as shown in Fig 3. According to the finite analytic method, the coefficients of the governing equation are constant in every small region. Therefore, the finite analytic solution of velocity in the small region [*Y*_*j*-1_, *Y*_*j*+1_] can be obtained as follows:

$$U_{j}=C_{j}e^{p_{j}Y}+D_{j}e^{q_{j}Y}+\frac{2}{\lambda_{j}}$$ (16)

where *p*_*j*_ and *q*_*j*_ are expressed as:

$$p_{j}=-\frac{\left[\begin{array}{l} 2n_{j}\zeta Y_{j}+Z_{j}+Z_{j}\zeta Y_{{}_{j}}^{2}+(Z_{j}^{2}\zeta^{2}Y_{j}^{4}+4n_{j}Z_{j}\zeta^{2}Y_{j}^{3}+4n_{j}^{2}\zeta^{2}Y_{{}_{j}}^{2}\\ +2Z_{j}^{2}\zeta Y_{{}_{j}}^{2}+4n_{j}Z_{j}\zeta Y_{j}+4n_{j}^{2}\lambda \zeta Y_{{}_{j}}^{2}+Z_{j}^{2}+4n_{j}^{2}\lambda {)}^{1/2} \end{array}\right]}{2n_{j}(1+\zeta Y_{{}_{j}}^{2})}$$ (17)

$$q_{j}=-\frac{\left[\begin{array}{l} 2n_{j}\zeta Y_{j}+Z_{j}+Z_{j}\zeta Y_{{}_{j}}^{2}-(Z_{j}^{2}\zeta^{2}Y_{j}^{4}+4n_{j}Z_{j}\zeta^{2}Y_{j}^{3}+4n_{j}^{2}\zeta^{2}Y_{{}_{j}}^{2}\\ +2Z_{j}^{2}\zeta Y_{{}_{j}}^{2}+4n_{j}Z_{j}\zeta Y_{j}+4n_{j}^{2}\lambda \zeta Y_{{}_{j}}^{2}+Z_{j}^{2}+4n_{j}^{2}\lambda {)}^{1/2} \end{array}\right]}{2n_{j}(1+\zeta Y_{{}_{j}}^{2})}$$ (18)

where Zj=(dndY)j.

For *j* ∈ [2, *jm* − 2], *C*_*j*_, *D*_*j*_ can be determined by point velocities of the adjacent boundary

$$C_{j}=\frac{\left(U_{j-1}-\frac{2}{\lambda_{j}}\right)e^{q_{j}Y_{j+1}}-\left(U_{j+1}-\frac{2}{\lambda_{j}}\right)e^{q_{j}Y_{j-1}}}{e^{p_{j}Y_{j-1}+q_{j}Y_{j+1}}-e^{p_{j}Y_{j+1}+q_{j}Y_{j-1}}}$$ (19)

$$D_{j}=\frac{\left(U_{j-1}-\frac{2}{\lambda_{j}}\right)e^{p_{j}Y_{j+1}}-\left(U_{j+1}-\frac{2}{\lambda_{j}}\right)e^{p_{j}Y_{j-1}}}{e^{p_{j}Y_{j+1}+q_{j}Y_{j-1}}-e^{p_{j}Y_{j-1}+q_{j}Y_{j+1}}}$$ (20)

(3) At the interface between the water layer and vegetation [*Y*_*jm* − 2_, *Y*_*jm*_], the coefficients of analytical solutions can be determined by Eqs (11), (12) and (15):

$$A=n_{jm}\left(C_{jm-1}e^{p_{jm-1}H_{v}}+D_{jm-1}e^{q_{jm-1}H_{v}}+\frac{2}{\lambda_{jm}}\right)+\frac{\text{ln}(\zeta H_{v}^{2}+1)}{\zeta }-\frac{2\text{arctan}(\sqrt{\zeta }H_{v})}{\sqrt{\zeta }}$$ (21)

B=−2 (22)

$$C_{jm-1}=\frac{\left(U_{jm-2}-\frac{2}{\lambda_{jm-1}}\right)q_{jm-1}e^{q_{jm-1}Y_{jm}}-\frac{2\left(1-Y_{jm}\right)}{\zeta Y_{jm}^{2}+1}e^{q_{jm-1}Y_{jm-2}}}{q_{jm-1}e^{q_{jm-1}Y_{jm}+P_{jm-1}Y_{jm-2}}-p_{jm-1}e^{P_{jm-1}Y_{jm}+q_{jm-1}Y_{jm-2}}}$$ (23)

$$D_{jm-1}=\frac{\frac{2\left(1-Y_{jm}\right)}{\zeta Y_{jm}^{2}+1}e^{p_{jm-1}Y_{jm-2}}-\left(U_{jm-2}-\frac{2}{\lambda_{jm-1}}\right)p_{jm-1}e^{p_{jm-1}Y_{jm}}}{q_{jm-1}e^{q_{jm-1}Y_{jm}+P_{jm-1}Y_{jm-2}}-p_{jm-1}e^{q_{jm-1}Y_{jm-2}+P_{jm-1}Y_{jm}}}$$ (24)

Thus, velocity can be determined by solving simultaneous equations in the vegetation area [*Y*_1_, *Y*_*jm*_].

As a special case, the velocity distribution of emergent vegetation flow (*H*_*v*_ = 1) can be obtained with the above method.

## Determination of permeability

Permeability is related to porosity *n*, tortuosity *T*_0_ (the ratio of actual length of flow path to the straight length along the macroscopic press gradient), and cross-sectional shape. Tortuosity is not a constant but dominated by porosity.

Many investigations have obtained different forms and laws of the expression of permeability *k* with the extension of porous media theory to other fields. Xu et al. [10] have summarized some modifications of the permeability. However, there is no expression of permeability for turbulent flow through submerged vegetation, which limits the application of the poroelastic media flow theory in vegetation flow.

**Fig 4:**
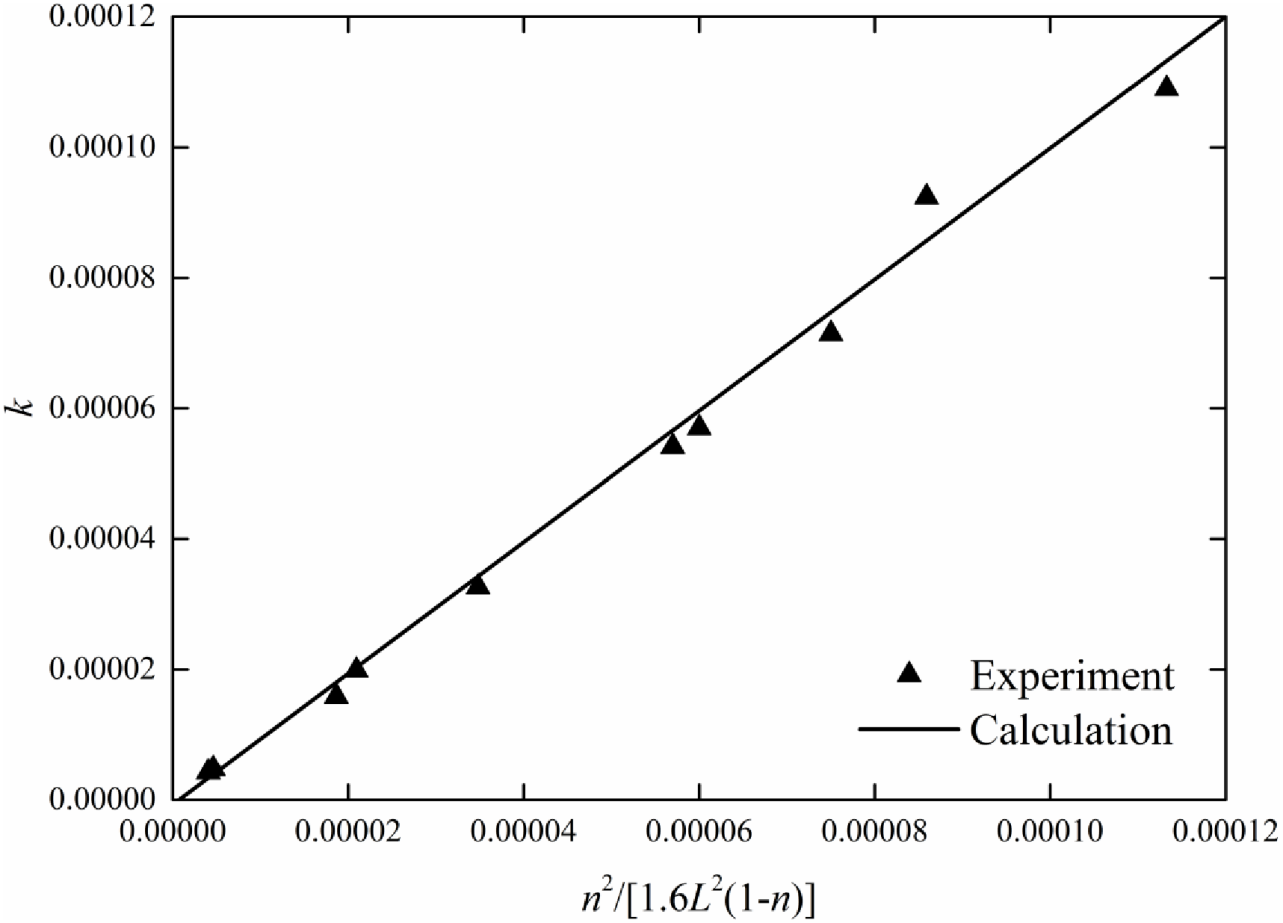
Fit of permeability in submerged vegetation flow.

By comparing the calculated velocity distribution having constant vertical porosity with the measured velocity distribution obtained by Shimizu Y et al. [15], Ghisalberti et al. [1] and Huai et al. [16], the best fitting expression for permeability is

$$k=\frac{n^{2}}{1.6L^{2}(1-n)}$$ (25)

where *L* is the ratio of the cross-sectional perimeter to the area. The result shows that permeability in turbulent flow through submerged vegetation varies with vegetation porosity and vegetation shapes. The fitting of permeability is shown in Fig 4.

For circular cross-sections, *L* is expressed as:

$$L=2\sqrt{\frac{\pi \alpha }{1-n}}$$ (26)

According to Eqs (25) and (26), the value of *λ* for non-constant vertical porosity is:

$$\lambda =\frac{6.48\pi \alpha h^{2}}{n}$$ (27)

## Experimental methods

**Fig 5:**
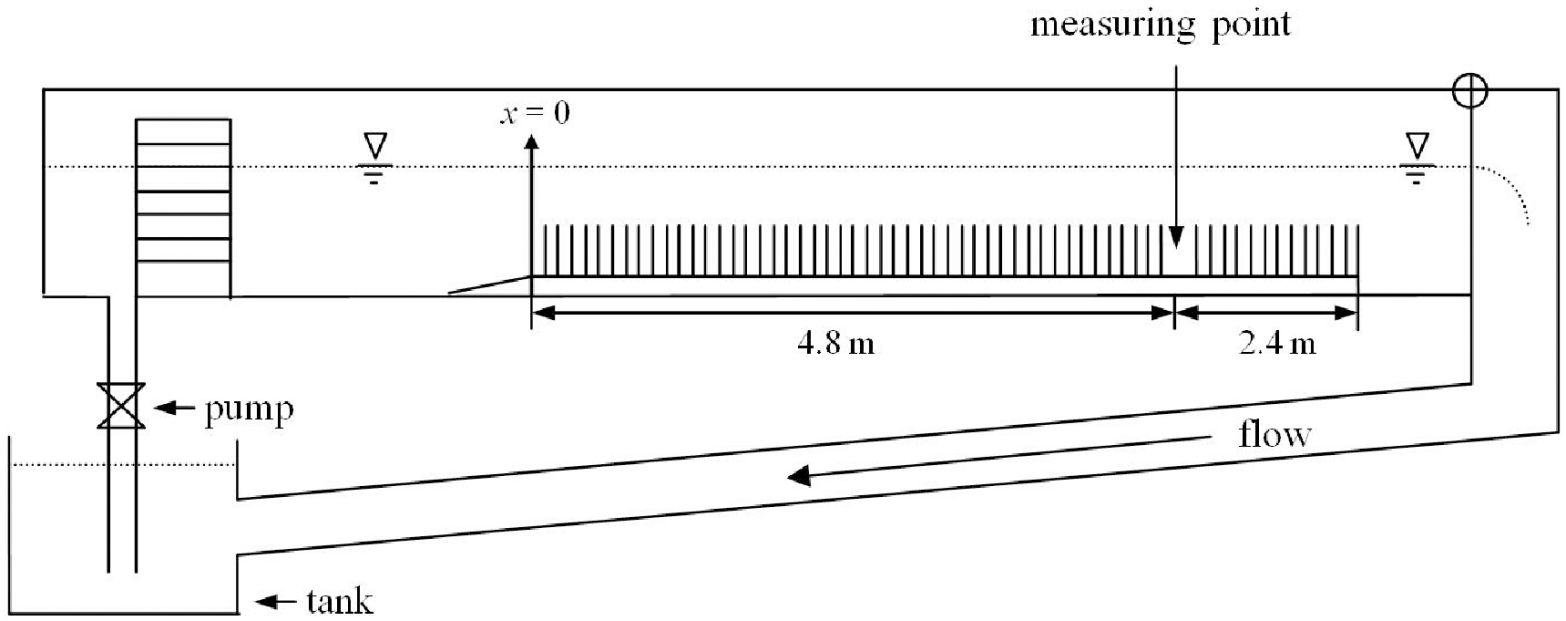
Layout of experimental installation.

The experiments are carried out in a straight and glass-walled flume of the State Key Laboratory of Water Resources and Hydropower Engineering Science at Wuhan University. Fig 5 shows the layout of the experimental installation. The flume is 1 m wide and 20 m long. The positive *x*-axis is in the stream-wise direction and the *y*-axis is in the cross-channel direction. The inlet and outlet of the flume are connected to a hydraulic circuit to ensure the continuous recirculation of stable discharges. There is an electronic flow meter at the inlet to measure water discharge. The water surface is adjusted with a tail-gate for uniform flow.

**Fig 6:**
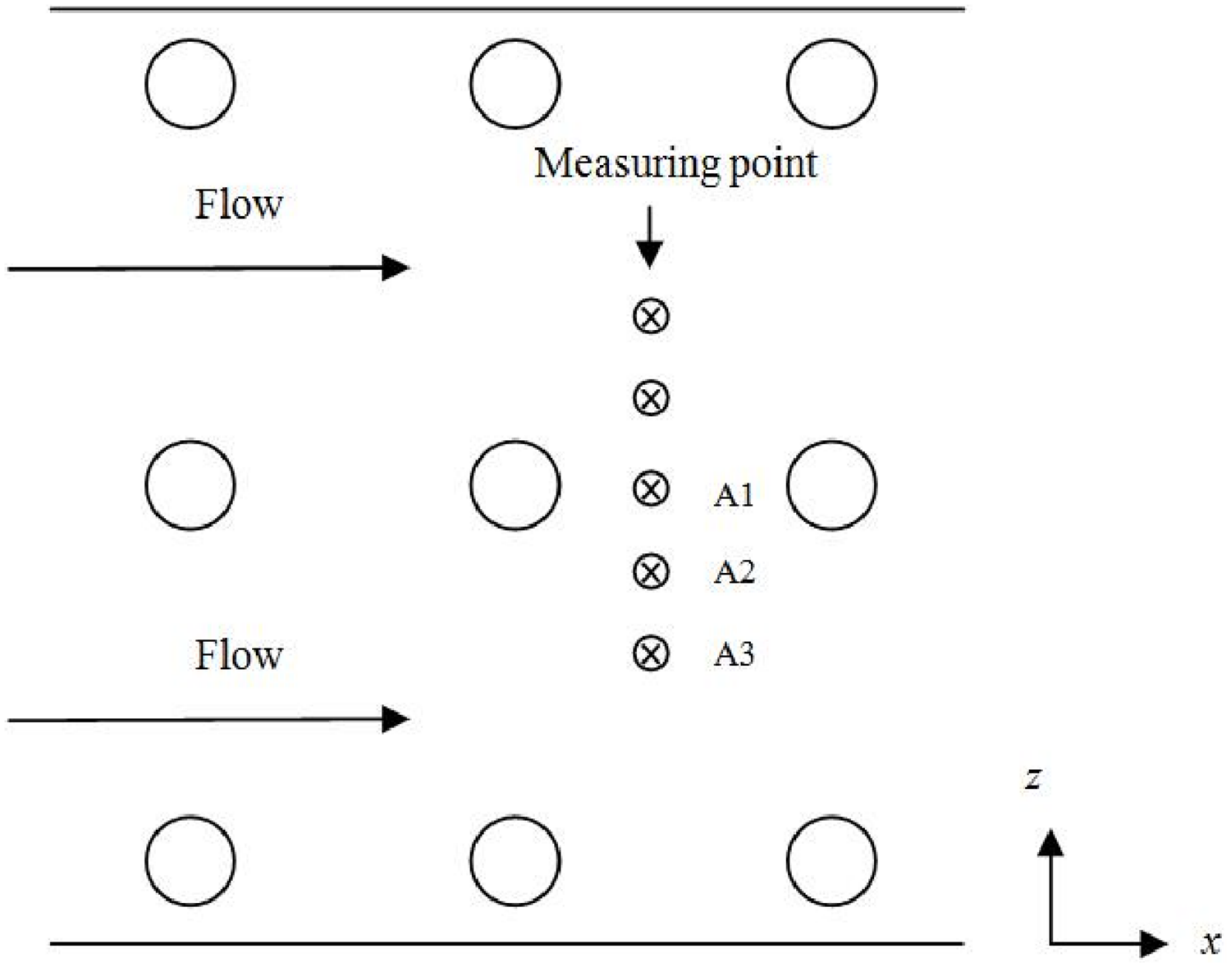
Plan view of the channel bed.

Wooden truncated cones (*r*_*max*_ = 0.4 cm and *r*_*min*_ = 0.2 cm) are arranged regularly in holes drilled into 0.5-m-long polyvinyl chloride boards. Twelve boards are used to create a 7.2-m-long vegetation zone model. Water depth is maintained at 36 cm. The height of the truncated cone is 24 cm. Vertical porosity is in the range of 0.982 to 0.996. Velocity measurements are taken simultaneously by three-dimensional acoustic Doppler velocimeters (ADV) equipped with an upward probe and a downward probe. All the ADV probes are placed at *x* = 4.8 m, and fully developed flow is established well before the sampling point. Five sampling points are located laterally behind every model plant stem to obtain the spatial mean velocity shown in Fig 6. The distance between adjacent measuring points is 1 cm. The experimental data are recorded at 50 Hz for 2 minutes at every position to collect 6000 data.

## Results and discussion

### Turbulence model

**Fig 7:**
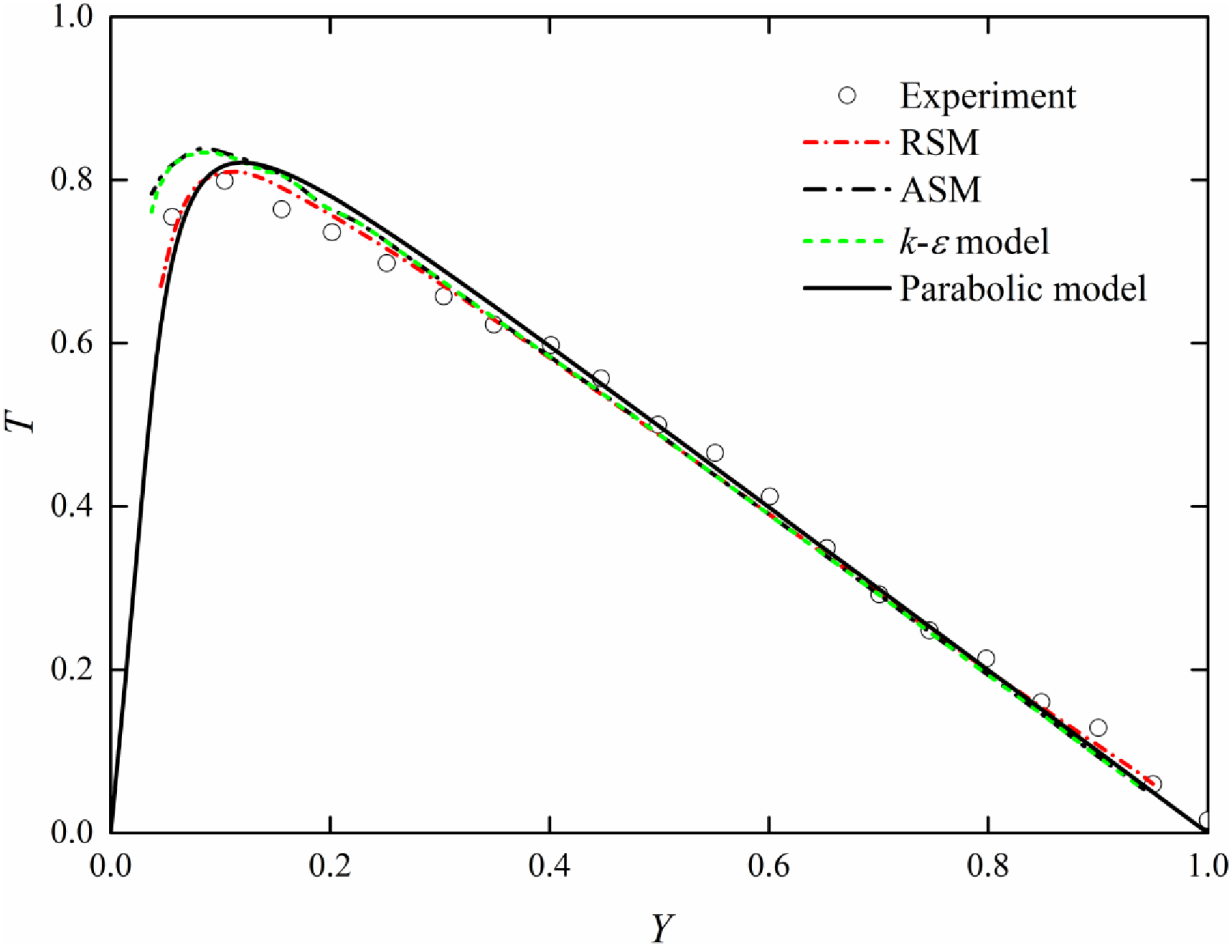
Comparison of different turbulence models and experimental data.

In order to verify the accuracy and rationality of the above turbulence model, *k*–*ε* model, algebraic stress model, and Reynolds stress model (Choi et al. [17]) are used for comparison. In the numerical simulation, the water depth is 0.05 m, the side slope is 1/6000, and the fitting value of turbulence coefficient *β* is 0.077 m^2^/s. The comparison between the computed Reynolds sheer stress and experimental data (Nezu et al. [18]) is shown in Fig 7 where *T* = *τ*_2_ / *ρghs*. It is obvious that all the computed Reynolds sheer stress distributions are nearly same and are in good agreement with experimental data, which indicates that the parabolic turbulence model has excellent accuracy.

### Constant vertical vegetation porosity

Constant vegetation porosity is a special case of this model; therefore, velocity distribution can be also obtained with the finite analytic method. Three sets of experimental data reported by Ghisalberti et al. [1] are used for verification.

In the experiment by Ghisalberti et al. [1], the constant water depth was 46.7 cm and the glass flume width was 38 cm. Canopies were simulated with circular wooden cylinders (*r* = 0.32 cm) arranged randomly on Plexiglas boards. Three packing densities were 0.025, 0.034, and 0.080 cm^-1^. The values of vegetation porosity were 0.987, 0.983, and 0.960. The canopy height was 13.8 or 13.9 cm. Due to the limitation of three-dimensional acoustic Doppler velocimeters; the uppermost 7 cm of the flow could not be sampled. The experimental parameters are listed in Table 1.

**Table 1:** Test conditions and calculated parameters.

Run	*Q* (l/s)	*h*_*v*_ (cm)	*H* (cm)	*n*	*s*	*r* (cm)
B	1.7	13.9	46.7	0.987	1.8×10^−6^	0.32
C	7.4	13.9	46.7	0.983	2.5×10^−5^	0.32
H	14.3	13.8	46.7	0.960	1.0×10^−4^	0.32
F1	37	24.0	36.0	0.982–0.996	5.2×10^−5^	0.2–0.4
F2	43.3	24.0	36.0	0.982–0.996	5.8×10^−5^	0.2–0.4
F3	52	24.0	36.0	0.982–0.996	7.4×10^−5^	0.2–0.4

**Fig 8:**
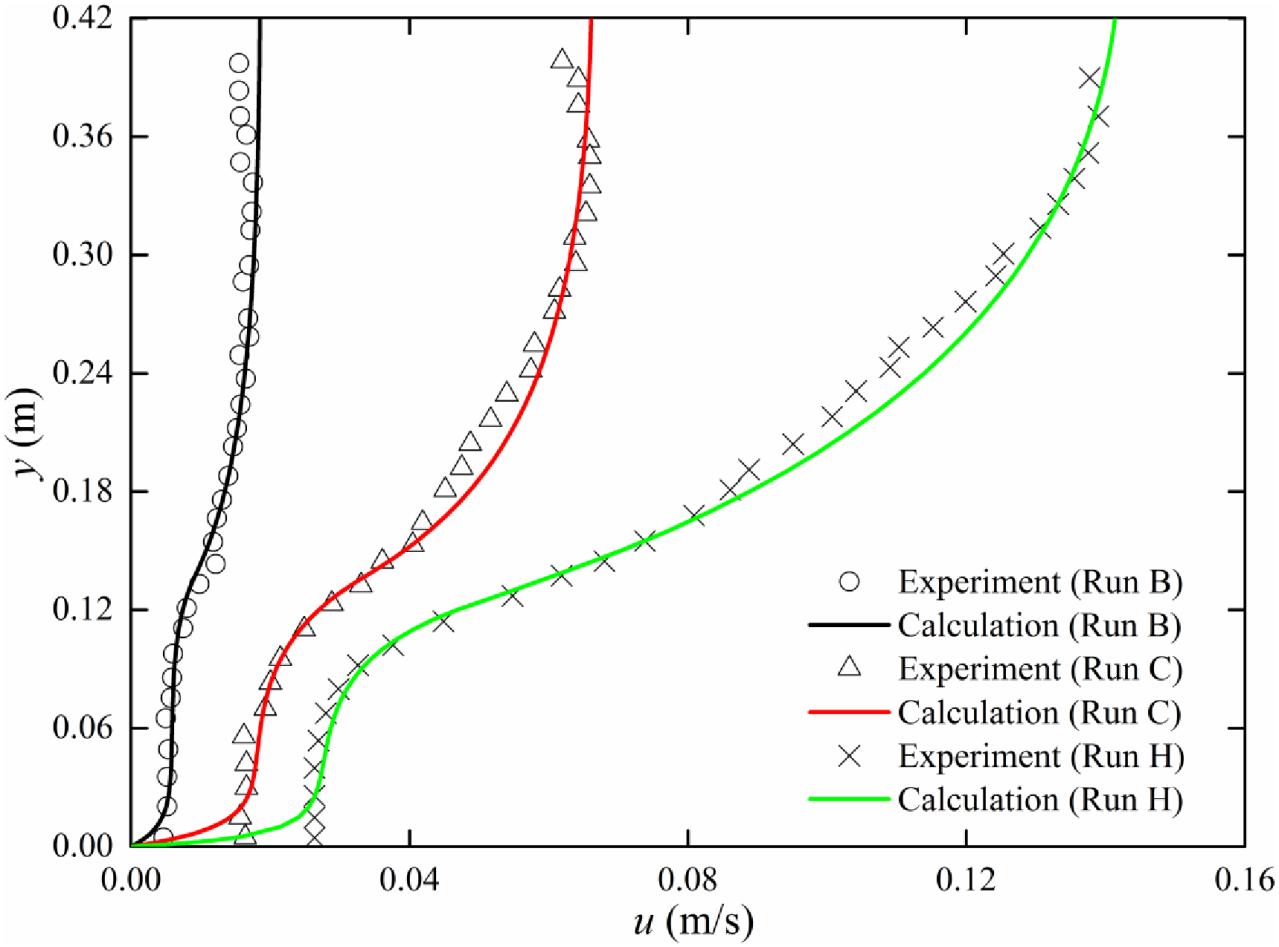
Comparison of measurement and finite analytic results (constant porosity).

Fig 8 shows the comparison of velocity distribution for constant porosity. It can be seen obviously that the theoretical results are in good agreement with experimental data. Therefore, the model can predict the vertical velocity distribution of vegetation flow with constant vertical porosity.

### Non-constant vertical vegetation porosity

**Fig 9:**
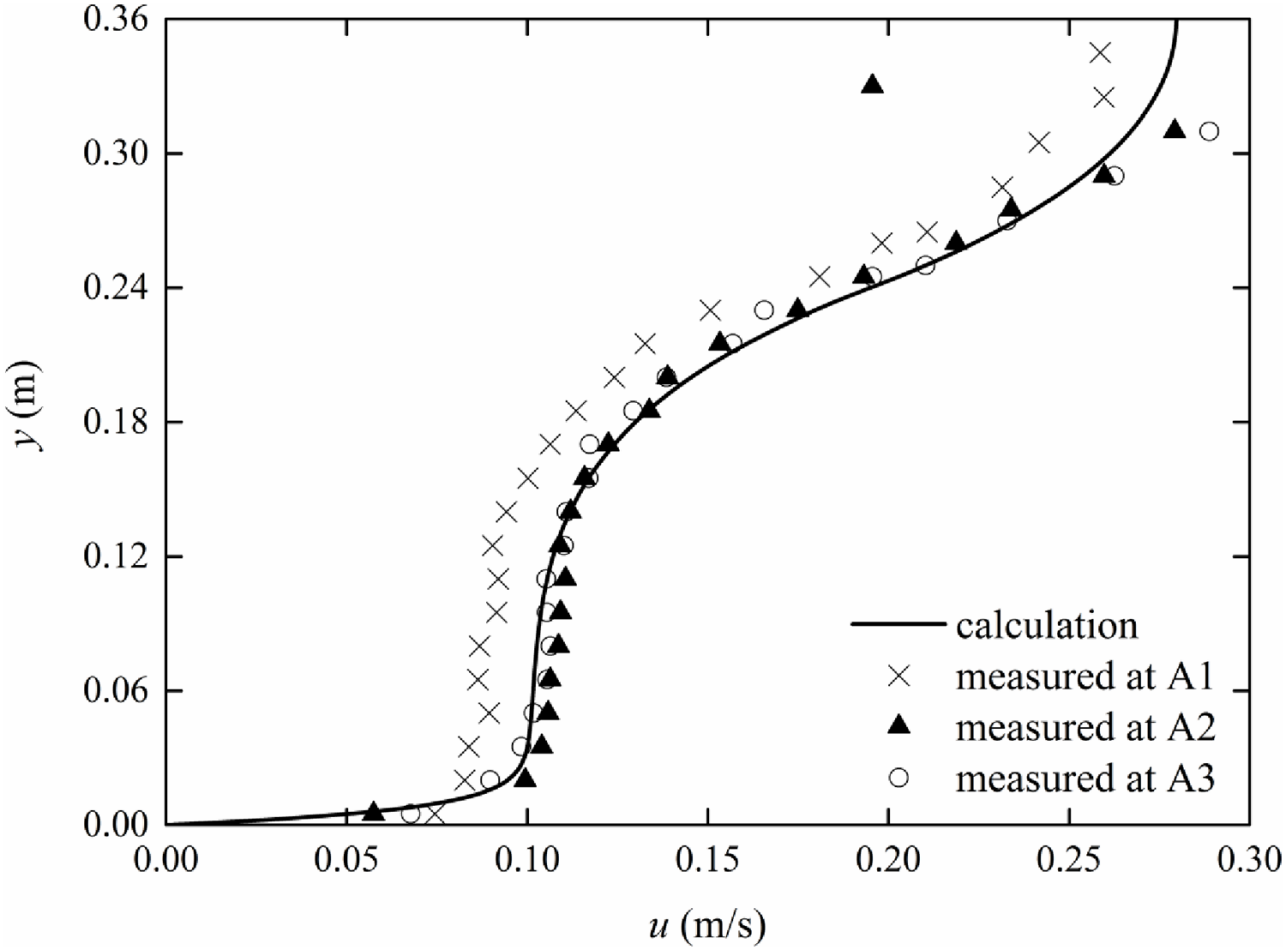
Comparison of measurement and finite analytic results (*Q* = 52.0 l/s).

**Fig 10:**
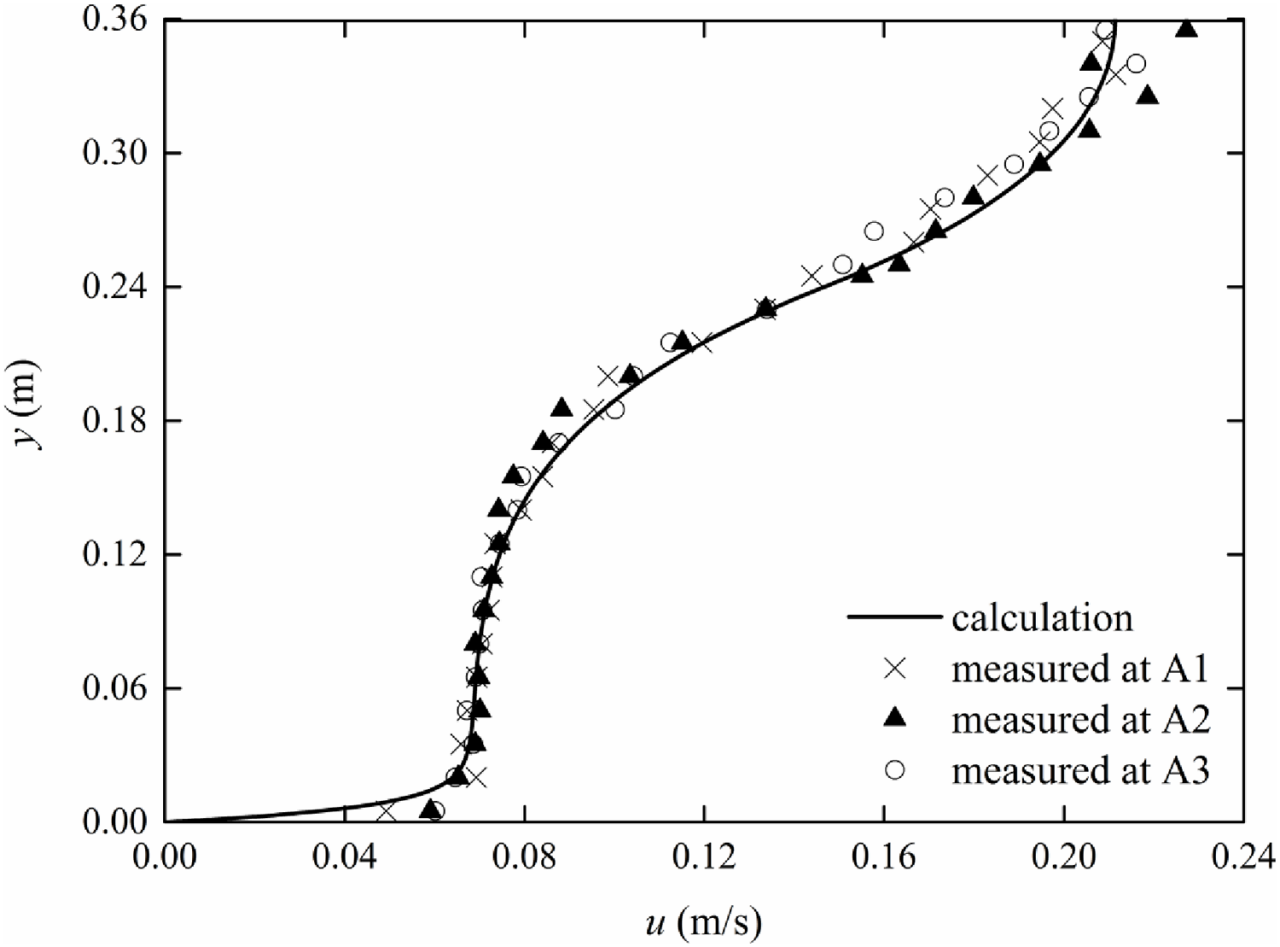
Comparison of measurement and finite analytic results (*Q* = 43.3 l/s).

**Fig 11:**
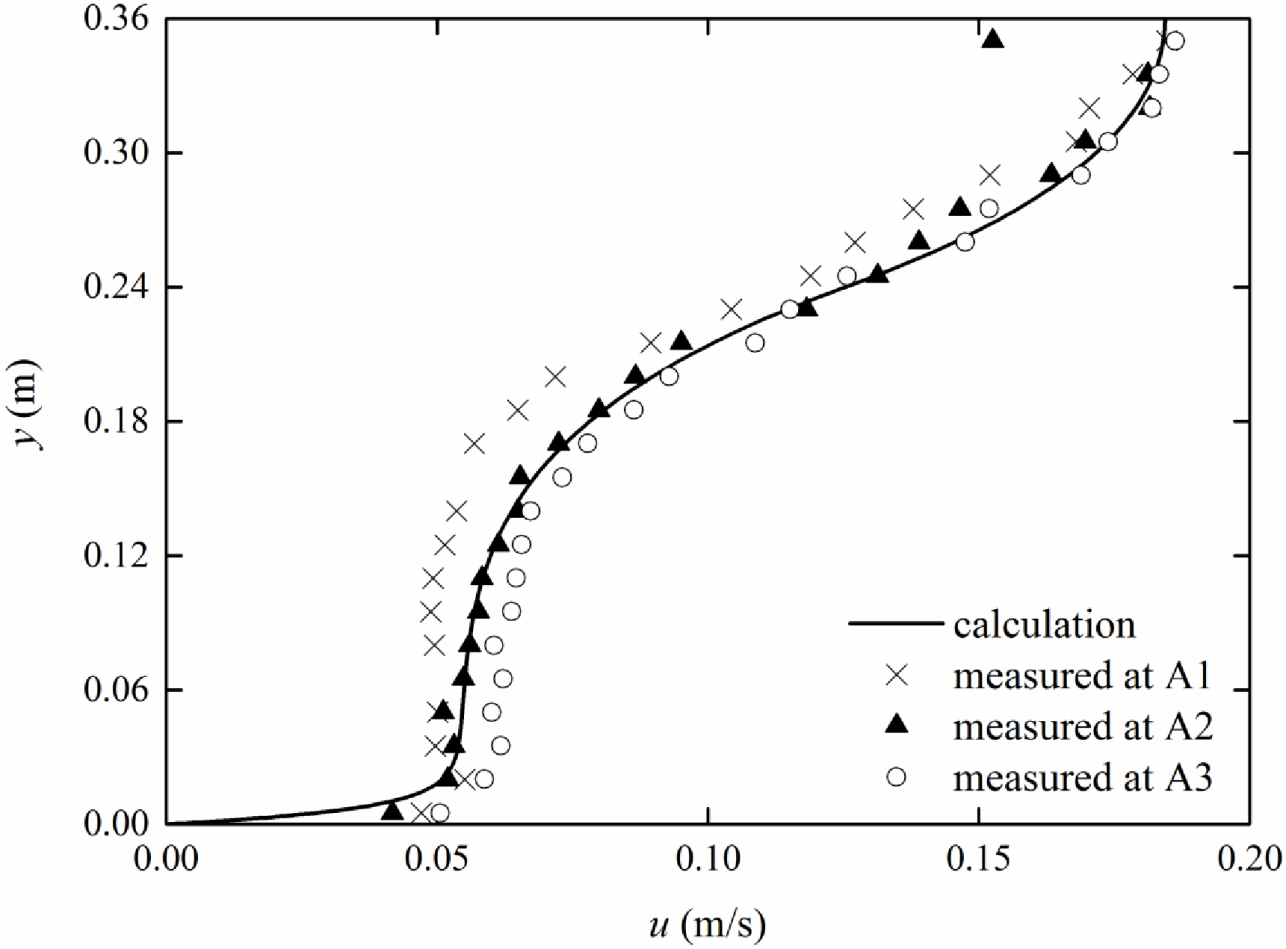
Comparison of measurement and finite analytic results (*Q* = 37.0 l/s).

The experimental parameters are listed in Table 1. In the experiment, rigid vegetation with non-constant vertical porosity is simulated with truncated cones with *r*_*min*_ = 0.2 cm and *r*_*max*_ = 0.4 cm. Therefore, according to Eq (3), the coefficients of the porosity equation can be obtained as *c*_1_ = -0.010, *c*_2_ = 0.028, and *c*_3_ = 0.982. By substituting Eqs (3) and (27) into Eq (9), the velocity distribution can be obtained with the finite analytic method. The comparison between experimental data and calculated results of the mean velocity distribution with non-constant vertical porosity is shown in Figs 9–11. The experimental data are listed in S1–S3 Tables.

From Figs 9–11, it can be seen that the calculated results are in good agreement with the measured data, which demonstrates that the theoretical equation can predict the velocity distribution of submerged vegetation flow with non-constant vertical porosity. The velocity continuously decreases as the measuring point moves from A1 to A3; this behavior may be caused by Flow around Cylinders. The ratio of the turbulent coefficients in the water layer and vegetation layer increases from 1.57 to 1.77 as the discharges increase from 37.0 l/s to 52 l/s. Thus, the turbulent coefficient *β* can be determined from experiments.

### Engineering application

The purpose of river ecological restoration is to restore the function of the river system, so as to restore the health of the river system [19–22]. Vegetation restoration is an important technique for river restoration. By affecting flow velocity and the sediment deposition, aquatic plants have a great impact on the river [23–25]. At the same time, the reasonable distribution of vegetation in flow is also helpful to reduce the flood disaster and purify sewage [26–31]. Due to considering the influence of varying vertical porosity, the formula presented in this paper can predict velocity distribution more practically and accurately. Even if aquatic plants have complex shapes, the velocity distribution can also be obtained with the finite analytic method. The practical and accurate velocity distribution is essential for simulation of flood and pollutant transport through submerged vegetation. Therefore, the new governing equation and approach can provide a useful reference for river ecological restoration.

## Conclusions

This paper studied the influence of non-constant vertical porosity on open channel flow with submerged vegetation and obtained the following conclusions.

(1) The new governing equation for the vegetation flow with non-constant vertical porosity is presented by applying the poroelastic media flow theory. The governing equation has a wide applicability. For *n* = 1, the governing equation can be simplified to solve the velocity in open channel without vegetation. For constant vertical porosity *n*, the analytical solution of velocity can also be obtained by the governing equation. For *H*_*v*_ = 1, the equations can be used in emergent vegetation flow.

(2) The fitting equation of permeability *k* in turbulent flow with submerged vegetation is derived by comparison with published experimental data. The fitting equation has a high accuracy when vegetation porosity ranges from 0.9 to 1.0. However, for a wider range of vegetation porosity, the accuracy of the fitting equation of permeability should be tested by future detailed investigation.

(3) The finite analytic solution of velocity is obtained with the finite analytic method which has high calculation accuracy.

(4) To study the influence of non-constant vertical porosity on vegetation flow, truncated cones are selected to simulate rigid vegetation. The experimental data of velocity in open channel flow with submerged vegetation are obtained via the flume experiment. The theoretical results are in good agreement with experimental data, which indicates that the model can predict the vertical velocity distribution of vegetation flow with variable vertical porosity.

(5) Velocity distribution varies with vertical porosity in the vegetation region. As porosity increases with water depth, the velocity in the middle region of vegetation layer also increases.

(6) For river ecological restoration, by considering the influence of vertical variation of porosity on velocity distributions, the model predicts the vertical distributions of stream-wise velocity more accurately. An accurate description of these flow variables is essential for modeling sediment transport, pollutant removal, flood control, and even the vegetation growth itself.

## Supporting information

S1 Table Experimental data for Run F1 (*Q* = 37.0 l/s). (DOCX)

S2 Table Experimental data for Run F2 (*Q* = 43.3 l/s). (DOCX)

S3 Table Experimental data for Run F3 (*Q* = 52.0 l/s). (DOCX)
