# Supplementary material for: Hydrodynamic characteristics of submerged vegetation flow with non-constant vertical porosity
Source: PLoS One. 2017 Apr 27;12(4):e0176712. doi: 10.1371/journal.pone.0176712 (PMC5407779; doi:10.1371/journal.pone.0176712)
Supplement: S1 Table — (DOCX) [file pone.0176712.s001.docx]

**S1 Table. Experimental data for Run F1 (*Q* = 37.0 l/s)**

| Measure at A1 | | Measure at A2 | | Measure at A3 | |
| --- | --- | --- | --- | --- | --- |
| *y* (m) | *u* (m/s) | *y* (m) | *u* (m/s) | *y* (m) | *u* (m/s) |
| 0.005 | 0.0471 | 0.005 | 0.04172 | 0.005 | 0.05054 |
| 0.020 | 0.05513 | 0.020 | 0.05192 | 0.020 | 0.05873 |
| 0.035 | 0.04969 | 0.035 | 0.05313 | 0.035 | 0.06178 |
| 0.050 | 0.05007 | 0.050 | 0.05112 | 0.050 | 0.06011 |
| 0.080 | 0.04952 | 0.065 | 0.05485 | 0.065 | 0.06219 |
| 0.095 | 0.04884 | 0.080 | 0.05599 | 0.080 | 0.06048 |
| 0.110 | 0.04927 | 0.095 | 0.05765 | 0.095 | 0.06373 |
| 0.125 | 0.05147 | 0.110 | 0.05825 | 0.110 | 0.06463 |
| 0.140 | 0.05364 | 0.125 | 0.06130 | 0.125 | 0.06564 |
| 0.170 | 0.05689 | 0.140 | 0.06490 | 0.140 | 0.06725 |
| 0.185 | 0.06490 | 0.155 | 0.06531 | 0.155 | 0.07312 |
| 0.200 | 0.07186 | 0.170 | 0.07242 | 0.170 | 0.07781 |
| 0.215 | 0.08948 | 0.185 | 0.07987 | 0.185 | 0.08635 |
| 0.230 | 0.10432 | 0.200 | 0.08661 | 0.200 | 0.09286 |
| 0.245 | 0.11898 | 0.215 | 0.09508 | 0.215 | 0.10870 |
| 0.260 | 0.12717 | 0.230 | 0.11824 | 0.230 | 0.11514 |
| 0.275 | 0.13795 | 0.245 | 0.13133 | 0.245 | 0.12570 |
| 0.290 | 0.15206 | 0.260 | 0.13900 | 0.260 | 0.14753 |
| 0.305 | 0.16797 | 0.275 | 0.14654 | 0.275 | 0.15196 |
| 0.320 | 0.17039 | 0.290 | 0.16336 | 0.290 | 0.16881 |
| 0.335 | 0.17838 | 0.305 | 0.16963 | 0.305 | 0.17386 |
| 0.350 | 0.18467 | 0.320 | 0.18147 | 0.320 | 0.18200 |
| - | - | 0.335 | 0.18113 | 0.335 | 0.18333 |
| - | - | 0.350 | 0.15261 | 0.350 | 0.18621 |
