# Supplementary material for: Hydrodynamic characteristics of submerged vegetation flow with non-constant vertical porosity
Source: PLoS One. 2017 Apr 27;12(4):e0176712. doi: 10.1371/journal.pone.0176712 (PMC5407779; doi:10.1371/journal.pone.0176712)
Supplement: S2 Table — (DOCX) [file pone.0176712.s002.docx]

**S2 Table. Experimental data for Run F2 (*Q* = 43.3 l/s)**

| Measure at A1 | | Measure at A2 | | Measure at A3 | |
| --- | --- | --- | --- | --- | --- |
| *y* (m) | *u* (m/s) | *y* (m) | *u* (m/s) | *y* (m) | *u* (m/s) |
| 0.005 | 0.04918 | 0.005 | 0.05906 | 0.005 | 0.06015 |
| 0.020 | 0.06927 | 0.020 | 0.06524 | 0.020 | 0.06462 |
| 0.035 | 0.06586 | 0.035 | 0.06904 | 0.035 | 0.06859 |
| 0.050 | 0.06726 | 0.050 | 0.07007 | 0.050 | 0.06715 |
| 0.065 | 0.06938 | 0.065 | 0.06979 | 0.065 | 0.06911 |
| 0.080 | 0.07056 | 0.080 | 0.06902 | 0.080 | 0.06996 |
| 0.095 | 0.0722 | 0.095 | 0.07098 | 0.095 | 0.07065 |
| 0.110 | 0.07271 | 0.110 | 0.07269 | 0.110 | 0.07039 |
| 0.125 | 0.07345 | 0.125 | 0.07436 | 0.125 | 0.07446 |
| 0.140 | 0.07925 | 0.140 | 0.07421 | 0.140 | 0.07840 |
| 0.155 | 0.08403 | 0.155 | 0.07748 | 0.155 | 0.07929 |
| 0.170 | 0.08617 | 0.170 | 0.08413 | 0.170 | 0.08764 |
| 0.185 | 0.09541 | 0.185 | 0.08827 | 0.185 | 0.10016 |
| 0.200 | 0.09855 | 0.200 | 0.10346 | 0.200 | 0.10424 |
| 0.215 | 0.11954 | 0.215 | 0.11517 | 0.215 | 0.11244 |
| 0.230 | 0.13355 | 0.230 | 0.13369 | 0.230 | 0.13398 |
| 0.245 | 0.14402 | 0.245 | 0.15517 | 0.250 | 0.15088 |
| 0.260 | 0.16665 | 0.250 | 0.16339 | 0.265 | 0.15784 |
| 0.275 | 0.17037 | 0.265 | 0.17156 | 0.280 | 0.17355 |
| 0.290 | 0.18307 | 0.280 | 0.17993 | 0.295 | 0.18901 |
| 0.305 | 0.19464 | 0.295 | 0.19470 | 0.310 | 0.19677 |
| 0.320 | 0.19746 | 0.310 | 0.20564 | 0.325 | 0.20555 |
| 0.335 | 0.21149 | 0.325 | 0.21858 | 0.340 | 0.21608 |
| 0.350 | 0.20854 | 0.340 | 0.20603 | 0.355 | 0.20926 |
