# Supplementary material for: Hydrodynamic characteristics of submerged vegetation flow with non-constant vertical porosity
Source: PLoS One. 2017 Apr 27;12(4):e0176712. doi: 10.1371/journal.pone.0176712 (PMC5407779; doi:10.1371/journal.pone.0176712)
Supplement: S3 Table — (DOCX) [file pone.0176712.s003.docx]

**S3 Table. Experimental data for Run F3 (*Q* = 52.0 l/s)**

| Measure at A1 | | Measure at A2 | | Measure at A3 | |
| --- | --- | --- | --- | --- | --- |
| *y* (m) | *u* (m/s) | *y* (m) | *u* (m/s) | *y* (m) | *u* (m/s) |
| 0.005 | 0.07440 | 0.005 | 0.05740 | 0.005 | 0.06770 |
| 0.020 | 0.08272 | 0.020 | 0.09943 | 0.020 | 0.08972 |
| 0.035 | 0.08382 | 0.035 | 0.10403 | 0.035 | 0.09837 |
| 0.050 | 0.08948 | 0.050 | 0.10575 | 0.050 | 0.10178 |
| 0.065 | 0.08631 | 0.065 | 0.10631 | 0.065 | 0.10552 |
| 0.080 | 0.08677 | 0.080 | 0.10866 | 0.080 | 0.10640 |
| 0.095 | 0.09156 | 0.095 | 0.10920 | 0.095 | 0.10536 |
| 0.110 | 0.09199 | 0.110 | 0.11058 | 0.110 | 0.10524 |
| 0.125 | 0.09043 | 0.125 | 0.10903 | 0.125 | 0.11013 |
| 0.140 | 0.09426 | 0.140 | 0.11199 | 0.140 | 0.11086 |
| 0.155 | 0.10018 | 0.155 | 0.11586 | 0.155 | 0.11698 |
| 0.170 | 0.10636 | 0.170 | 0.12244 | 0.170 | 0.11730 |
| 0.185 | 0.11354 | 0.185 | 0.13377 | 0.185 | 0.12937 |
| 0.200 | 0.12413 | 0.200 | 0.13888 | 0.200 | 0.13855 |
| 0.215 | 0.13265 | 0.215 | 0.15338 | 0.215 | 0.15693 |
| 0.230 | 0.15070 | 0.230 | 0.17493 | 0.230 | 0.16562 |
| 0.245 | 0.18100 | 0.245 | 0.19326 | 0.245 | 0.1956 |
| 0.260 | 0.19827 | 0.260 | 0.21883 | 0.250 | 0.21034 |
| 0.265 | 0.21077 | 0.275 | 0.23406 | 0.270 | 0.23291 |
| 0.285 | 0.23157 | 0.290 | 0.25971 | 0.290 | 0.26256 |
| 0.305 | 0.24176 | 0.310 | 0.27924 | 0.310 | 0.28886 |
| 0.325 | 0.25969 | 0.330 | 0.19563 | 0.330 | 0.29001 |
